# A Flexible, Quantitative Plasmonic-Fluor Lateral Flow Assay for the Rapid Detection of *Orthoebolavirus zairense* and *Orthoebolavirus sudanense*

**DOI:** 10.1021/acsinfecdis.3c00423

**Published:** 2023-12-04

**Authors:** Abraham J. Qavi, Qisheng Jiang, M. Javad Aman, Hong Vu, Larry Zetlin, John M. Dye, Jeffrey W. Froude, Daisy W. Leung, Frederick Holtsberg, Scott L. Crick, Gaya K. Amarasinghe

**Affiliations:** † Department of Pathology and Laboratory Medicine, University of California, Irvine, Irvine, California 92697, United States; ‡ Auragent Bioscience, St. Louis, Missouri 63108, United States; § Integrated Biotherapeutics, Rockville, Maryland 20850, United States; ∥ Mapp Biopharmaceutical, Inc., San Diego, California 92121, United States; ⊥ United States Army Medical Research Institute of Infectious Diseases, Fort Detrick, Maryland 21702, United States; # United States Army Nuclear and Countering Weapons of Mass Destruction Agency, Fort Belvoir, Virginia 22060, United States; ∇ Department of Medicine, Washington University School of Medicine, St. Louis, Missouri 63110, United States; ⊗ Department of Pathology & Immunology, Washington University School of Medicine, St. Louis, Missouri 63110, United States

**Keywords:** Filoviruses, diagnostics, plasmonics, lateral flow assay

## Abstract

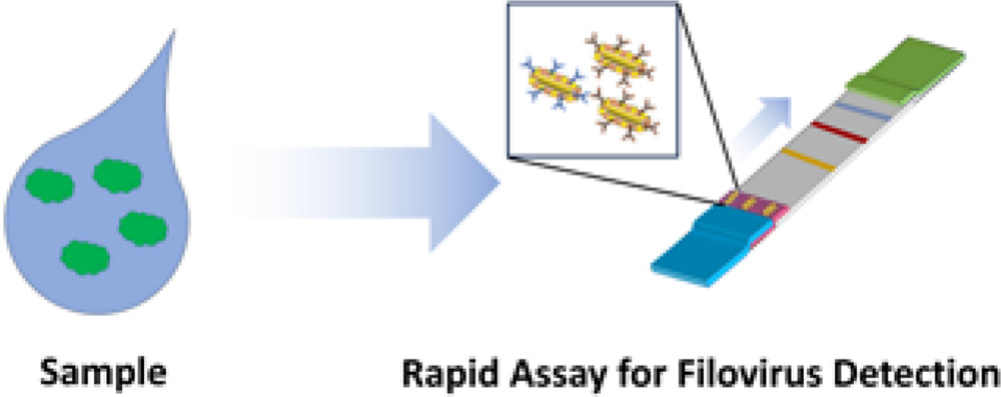

Filoviruses comprise a family of single-stranded, negative-sense RNA viruses with a significant impact on human health. Given the risk for disease outbreaks, as highlighted by the recent outbreaks across Africa, there is an unmet need for flexible diagnostic technologies that can be deployed in resource-limited settings. Herein, we highlight the use of plasmonic-fluor lateral flow assays (PF-LFA) for the rapid, quantitative detection of an Ebolavirus-secreted glycoprotein, a marker for infection. Plasmonic fluors are a class of ultrabright reporter molecules that combine engineered nanorods with conventional fluorophores, resulting in improved analytical sensitivity. We have developed a PF-LFA for *Orthoebolavirus zairense* (EBOV) and *Orthoebolavirus sudanense* (SUDV) that provides estimated limits of detection as low as 0.446 and 0.641 ng/mL, respectively. Furthermore, our assay highlights a high degree of specificity between the two viral species while also maintaining a turnaround time as short as 30 min. To highlight the utility of our PF-LFA, we demonstrate the detection of EBOV infection in non-human primates. Our PF-LFA represents an enormous step forward in the development of a robust, field-deployable assay for filoviruses.

Ebolavirus disease (EVD) is caused by members within the Filoviridae family, *Orthoebolavirus* genus, of which four species have been documented to cause disease within humans: *Orthoebolavirus zairense* (EBOV), *Orthoebolavirus sudanense* (SUDV), *Orthoebolavirus bundibugyoense* (BDBV), and *Orthoebolavirus taiense* (TAFV).^1^*O. zairense*, initially discovered in 1976, accounts for the greatest number of outbreaks and for the largest outbreak to date in the Democratic Republic of the Congo (DRC) in 2014, with over 28,000 individuals infected and over 11,000 dead.^2^ Consequently, efforts to mitigate outbreaks and advances in treatment have been primarily focused on *O. zairense*. As highlighted by the recent SUDV outbreak in Uganda, there is still a critical need for the development of new treatments and diagnostics for filoviruses other than EBOV.^3−6^

Current diagnostic paradigms for filoviruses rely on reverse transcriptase polymerase chain reaction (RT-PCR) and antigen-based detection techniques.^3,4^ While conventional RT-PCR is the gold standard for diagnosis, there are several constraints that limit its utility in field settings. Among these are the needs for consistent power supplies, trained laboratory technicians, and cold chain custody of reagents. Consequently, traditional RT-PCR techniques are often constrained to core laboratories. In contrast, antigen-based detection techniques are often integrated with lateral flow assays (LFAs). While these offer portability and ease of use, they are often binary in their readout, providing a positive or negative answer rather than a quantitative value. Moreover, they are typically quite insensitive, and currently available LFAs for filoviruses usually focus on the detection of viral protein 40 (VP40), nucleoprotein (NP), or glycoprotein (GP).^4^ These antigen-based markers are typically positive after RT-PCR,^7^ thereby limiting their diagnostic window and efficacy for screening purposes.

Herein, we describe the development of a LFA against EBOV and SUDV, leveraging plasmonic fluors (PFs), a class of ultrabright fluorescent labels. PFs are engineered gold and silver nanorods with conjugated fluorophores. The resulting fluor is brighter than conventional fluorophores, consequentially leading to reduced volume requirements, improved signal-to-noise, and improved analytical sensitivity. These probes have been detected previously in both plate-based array formats as well as LFAs for the detection of a variety of analytes.^8−11^ In addition to the unique readout of this assay, we make use of unique antibodies against the soluble glycoprotein (sGP) of SUDV and EBOV. Previous studies have suggested that sGP serves as a diagnostic and prognostics marker for *Orthoebolavirus* infection.^12 ,13^ Together, our PF-LFA along with the informative biomarkers for filoviral infection result in a highly sensitive assay with potential for use during filoviral outbreaks.

A schematic of the LFA developed in our study is highlighted in Figure 1. The PF-LFA consists of a sample pad, followed by a nitrocellulose membrane with capture antibodies printed on it and an absorption pad. Antibodies against SUDV sGP, EBOV sGP, and goat IgG (control) are arrayed sequentially. The sample of interest is either incubated in a solution containing pan-filoviral antibodies conjugated to PFs, after which the sample is added to the assay (Figure 1a), or directly applied to a PF-LFA containing the aforementioned antibody–PF conjugates (Figure 1b). Capillary action pulls the specimen across the LFA, in addition to specific filoviral antibodies conjugated to PFs as well as control antibodies. The specimen mixture is finally deposited into the absorption pad at the end of the LFA, and then the assay is read-out using a fluorescent reader developed in-house by Auragent Bioscience.^9^ The total dimensions of a single strip are 3 mm × 60 mm.

**Figure 1:**
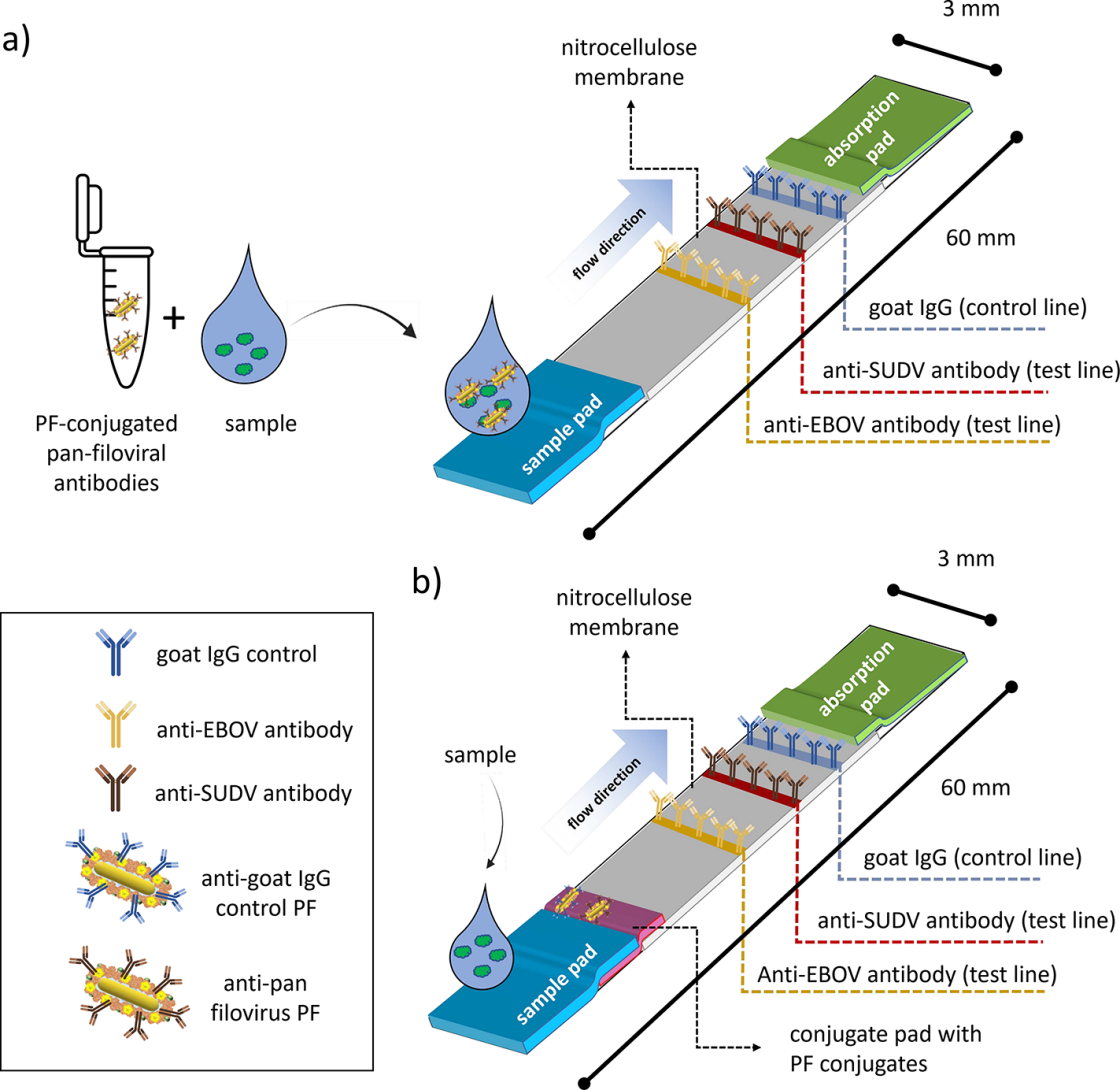
**Schematic of the plasmonic-fluor lateral flow assay (PF-LFA) 2-plex assay for the detection of EBOV and SUDV.** The PF-LFA can be operated (a) with a pre-incubation step or (b) by directly adding the sample of interest to a test strip. (a) The sample of interest is pre-incubated with a solution containing plasmonic fluors conjugated with pan-filoviral antibodies for 15 min. Subsequently, this mixture is added to the LFA via the sample pad. Capillary action moves the solution across the strip, toward the absorbent pad. The sample passes across stripes of antibodies against EBOV sGP, SUDV sGP, and a control, after which the signal is read via a fluorescent reader. (b) Alternatively, as shown in the schematic below, a conjugate pad containing the PF conjugates can be integrated into the device to eliminate pre-incubation.

To verify selectivity of the antibodies employed in our assay with the LFAs, we ran experiments with the detection of a single sGP target, either EBOV and SUDV, and examined the cross-reactivity with the off-target antibody at various concentrations of EBOV sGP and SUDV sGP and various incubation times. Figure S1-Sn highlights the responses for the on-target, off-target, and control at varying concentrations and incubation times. For both EBOV and SUDV capture antibodies, there is minimal cross-reactivity over the concentration ranges and pre-incubation times. These results are consistent with previous work with these antibody pairs.^12^ We also observed that running samples at a total concentration of 10% serum produced the most robust and sensitive results, as increasing the percentage serum decreased the analytical performance of the assay (Figure S2-Sn). For the pre-incubation model of our assay, we also observed that increasing the pre-incubation time improved both the estimated lower limit of detection and the working range of our assay (Figure 2).

**Figure 2:**
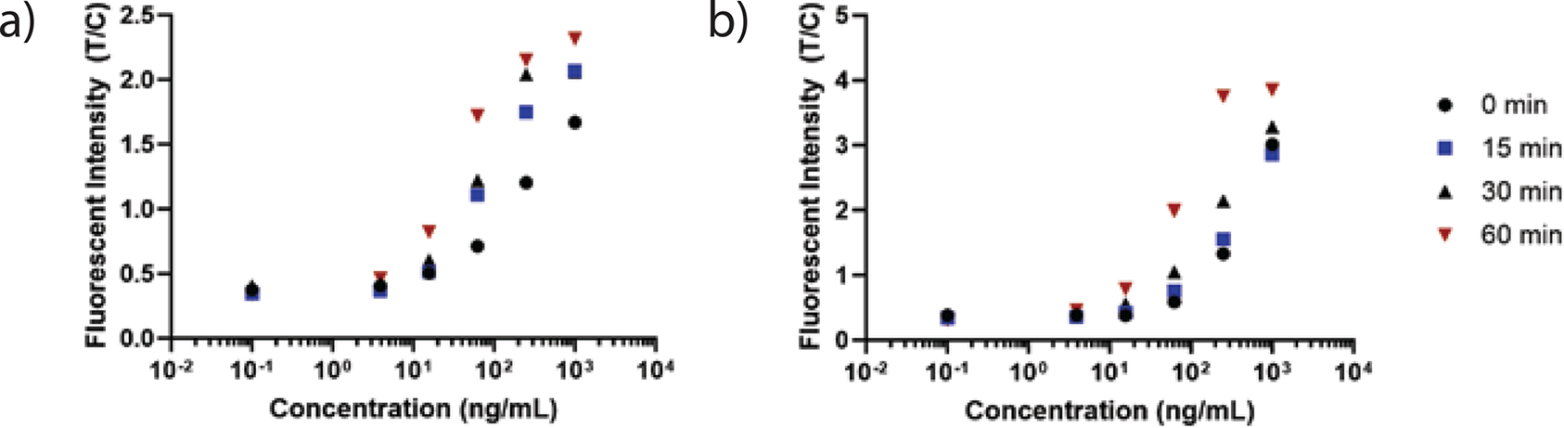
**Increased pre-incubation time results in improved assay performance.** For both (a) EBOV sGP and (b) SUDV sGP, increased pre-incubation time of the sample specimen with the pan-filoviral secondary antibodies conjugated to PFs resulted in an improvement of assay sensitivity, as well as an improvement in dynamic range. Each data point is *n* = 1.

Using spike-in studies in pooled human serum, we were able to quantify the analytical sensitivity and lower limit of detection of the PF-LFAs for both EBOV sGP and SUDV sGP (Figure 3a). With a pre-incubation time of 15 min with the sample and pan-filoviral antibodies, followed by a 15 min run-time for the LFA, the total assay time was 30 min. The estimated limits of detection for EBOV sGP and SUDV sGP were 0.446 and 0.641 ng/mL, respectively. When the sample was directly applied to the test strips (no pre-incubation period), our estimated limit of detection increased to 2.15 and 1.07 ng/mL for EBOV sGP and SUDV sGP, respectively (Figure S3-Sn). We observed that, at higher concentrations of the target, with both pre-incubation and direct sample application, a hook effect was present. In LFAs, this is commonly observed due to excess antigen, often leading to a paradoxical decrease in signal response.^14^

**Figure 3:**
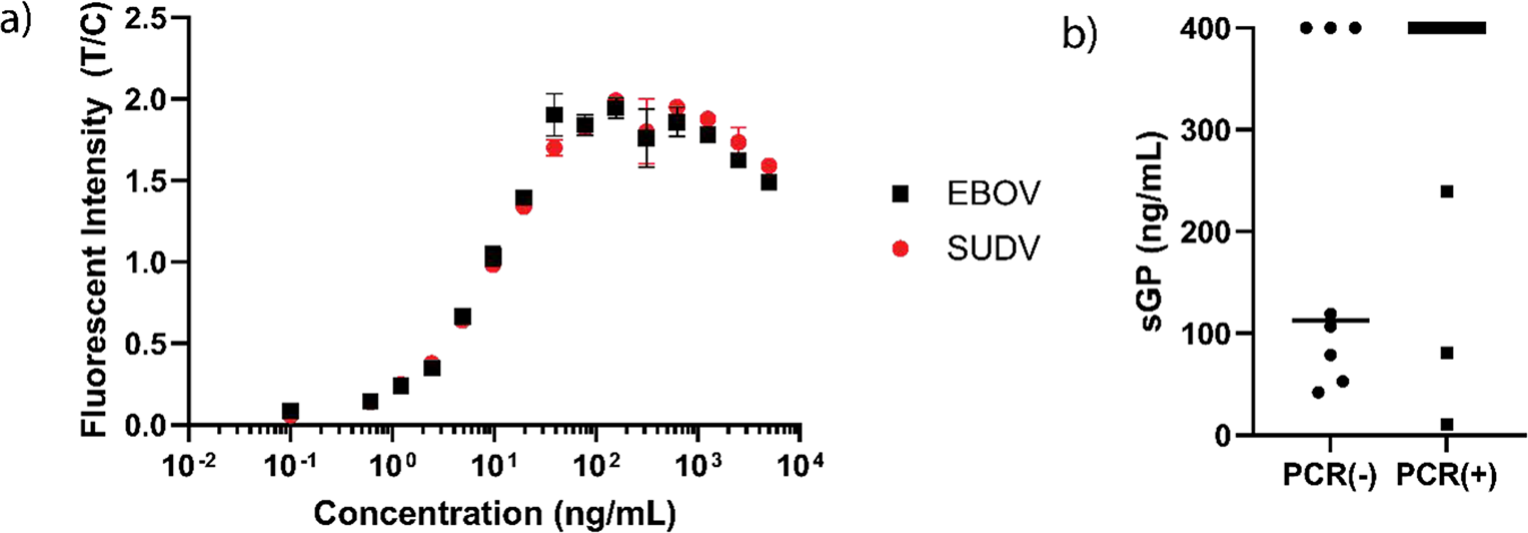
**Calibration curves for PF-LFAs against filoviral sGP and detection of sGP from EBOV-infected non-human primate (NHP) samples.** (a) Calibration curves for the detection of EBOV sGP and SUDV sGP in spiked human serum with our PF-LFAs. All samples were pre-incubated with PF–pan-filoviral antibodies for 15 min prior to running the assay. The lower limits of detection were 0.446 and 0.641 ng/mL for EBOV sGP and SUDV sGP, respectively. Error bars represent the standard deviations for *n* = 2. (b) Comparison of results for PF-LFA and RT-PCR on NHP samples infected with EBOV. All samples were pre-incubated with PF–pan-filoviral antibodies for 15 min prior to running the assay. RT-PCR detected a total of 22/30 (73.3%) of samples as positive, whereas the PF-LFA platform detected 30/30 (100%). Samples with a concentration value listed as 400 ng/mL had saturation of the fluorescent signal and therefore were considered positive. *n* = 3 for each of the samples, with the exception of Specimen #23 in which *n* = 1.

To validate the performance of our PF-LFA, we ran serum samples from EBOV-infected non-human primates (NHPs). Each sample was run identically to the calibration curves in Figure 3a—that is, a 15 min pre-incubation period followed by running the sample on the PF-LFA. Additionally, these are the same samples run in previous literature reports with a photonic resonator platform.^12^ While RT-PCR correctly identified infection in 22/30 samples (73.3%), the PF-LFA detected infection in 30/30 (100%) of the samples (Figure 3b).

Existing filoviral diagnostics typically rely on either nucleic acid amplification testing (NAAT) or antigen-based testing. There are several real-time, RT-PCR-based assays available on the market for EBOV, including those developed by government organizations (e.g., the DoD and CDC) and private companies (Cepheid, Altona Diagnostics GmbH, BioFire Defense). The majority of these assays focus on EBOV genes, namely, GP, L, NP, and VP40, although there are several RT-PCR assays that do specifically target filoviruses other than EBOV. While NAAT offers high analytical sensitivity, the needs for sample extraction (which comes with increased risk for unintentional exposure to specimens), uninterrupted power supplies, and technical expertise still limit these assays to core laboratory settings. The turnaround time for most NAAT-based assays is between 4 and 6 h, although BioFire and Cepheid’s platforms have been able to improve on this to as short as 75 min. Ultimately, this limits their ability to be rapidly deployed in field settings in developing economies. Loop-mediated isothermal amplification (LAMP) RT-PCR bypasses many of the limitations of traditional NAAT, as the lack of thermal cycling drastically simplifies the assay design and equipment requirements. LAMP-based techniques have been implemented as a simpler alternative to RT-PCR and have been demonstrated with patient samples.^15,16^ In contrast, antigen-based assays for filoviruses take advantage of LFAs, including the ReEBOV Antigen Rapid Test (Corgenix), the OraSure Ebola Rapid Antigen Test (Orasure Technologies), and SD Q Line Ebola Zaire Ag (SD Biosensor). LFAs are an attractive platform on which to develop rapid, point-of-care (POC) diagnostics due their ease of use, low cost, and rapid time to result.^17^ Conventional LFAs, such as those employed for pregnancy testing or SARS-CoV-2 rapid antigen tests, typically employ gold nanoparticles or latex beads with dyes as reporter molecules.^18^ The resultant readout signal is colorimetric and sufficient in many applications where a binary answer for infection is sufficient. A major disadvantage of LFA is its analytical sensitivity, which in turn can lead to poor clinical sensitivity. While these tests can offer a turnaround time between 15 and 30 min, the results are qualitative, and the assays can only target EBOV. While there is still significant work needed regarding prognostic markers of filoviral infection, the lack of quantitative information on these assays is a significant limitation.

In a resource-limited setting, the ability to accurately diagnose patients is critical for the effective allocation of healthcare resources and personnel.^19^ Our assay represents a critical step in addressing and curbing outbreaks by combining traditional LFA technology with PFs as the reporter molecules to create an ultrasensitive, quantitative, and rapid diagnostic test. As previously highlighted,^9^ PFs take advantage of precise engineering of silver and gold nanorods to enhance the brightness of fluorescent probes. This enhancement leads to a drastic increase in the analytical sensitivity. Previous work with PFs demonstrated an improvement of over 10^3^ relative to a more traditional colloidal gold LFA.^9^ When combined with a fluorescent reader, PF-LFA assays can provide both a highly sensitive and quantitative result. In combination with sGP, a biomarker that is both diagnostic and potentially prognostic, our assay provides an incredibly useful tool in combating filoviral outbreaks.

Another advantage of our assay is the target biomarker for filovirus infection, soluble glycoprotein (sGP). There are several proposed roles for sGP in the pathogenesis of EBOV, including as an immune decoy,^20,21^ for immunity modulation of the infected host,^22−24^ or for activation of host signaling pathways to augment uptake and internalization of the virus.^25^ Previous work has demonstrated that sGP levels rise concurrently or even prior to RT-PCR positivity during infection in NHP,^12^ which may allow for detection of filoviruses during the incubation period following infection.^26^ The early rise of sGP may account for the improved performance of our assay versus RT-PCR. In negative-sense, single-stranded RNA viruses, a positive-sense antigenome is used as a template to create genomic, negative-sense RNA. The necessary molecular components for transcription may further accentuate the lag between the presence of sGP and genome copies, necessary for RT-PCR amplification. This may account for the improved performance of our assay versus RT-PCR. Additionally, the limited sample preparation necessary with our assay might further improve its clinical sensitivity relative to RT-PCR, which requires RNA extraction.

Importantly, the rapid development and deployment of the diagnostic assay presented in this paper highlight one of the technology’s biggest strengths: the ability to rapidly adapt assays toward a variety of emerging and re-emerging pathogens. The simplicity and modularity of the PF-LFA assay design can be adapted for any existing sandwich pair and still utilize the PFs. As highlighted by the recent SUDV outbreak in Uganda, there is a paucity of analytical techniques for filoviruses other than EBOV.^6^ This represents a critical gap in our current arsenal of deployable diagnostics to address future filoviral outbreaks—one that we believe our PF-LFA platform addresses.

There are several challenges moving forward with our PF-LFA. For one, the results highlighted in our paper were conducted using a table-top reader, as previously described by Gupta and colleagues.^9^ To fully utilize our assay in the field, a portable reader is necessary that can operate without the need for uninterrupted power (e.g., one with an internal battery). The technical requirements for such a reader would also necessitate minimal moving parts, tolerance to environment variables in the field (e.g., temperature, humidity, and vibration control), the ability to run on an internal battery, a low target price, and a user interface and readout amenable for healthcare workers who may not have technical laboratory experience. Depending on where the assay is deployed, healthcare privacy concerns regarding reporting of the test results are to be considered as well.

Our study also focused on the use of serum, in part due to the feasibility of the proof-of-concept study. Whole blood, including capillary sticks, would require minimal sample preparation and would be a preferable specimen to incorporate into the PF-LFAs as it requires significantly less sample preparation and could be acquired without vacutainers and the need for centrifugation. A blood-to-serum separator pad can be incorporated into the PF-LFAs, enabling the use of whole blood such as that from finger sticks. The analytical performance of such an addition would undoubtedly affect the performance of our PF-LFAs and would require further optimization.

Another potential challenge with our assay is the presence of the hook effect—that is, the paradoxical decrease in signal observed at higher concentrations of sGP.^14^ This can be problematic if the assay is to be used for prognostication or monitoring trends of sGP levels in infected patients. One potential solution is to dilute samples from patients in which the levels of sGP are inconsistent with the clinical presentation. This may not always be obvious, especially given that the most common symptoms of EVD are nonspecific,^27^ and therefore further optimization of the PF-LFA will be required to minimize the hook effect for field applications. However, if our assay is to be used as a binary response for infection (e.g., yes or no), then the hook effect plays a less significant role.

In summary, the results of our work highlight the utility of PF-LFAs for the rapid, sensitive, and quantitative detection of EBOV and SUDV, with a low ng/mL sensitivity and rapid time to result. The flexible design of our assay, unique biomarker target, and potential to be deployed in the field in future iterations of the reader make our device appealing as a tool for combating future filoviral outbreaks. The next steps in the development of our assay will involve the optimization of our assay design to improve analytical sensitivity and fully assess cross-reactivity with a variety of pathogens and the implementation of a portable reader to fully deploy the assay in field settings.

## Methods

### Resource Availability

Requests for further information and for resources and reagents should be directed to the Lead Contact, Gaya Amarasinghe (gamarasinghe@wustl.edu). This study did not generate new unique reagents.

### Method Details

#### Capture Agents and Antigens

EBOV sGP (catalog no. 0565-001), SUDV sGP (catalog no. 0570-001), antibodies against EBOV sGP (catalog no. 0365-001), antibodies against SUDV sGP (catalog no. 0302-030), and pan-filoviral antibodies (catalog no. N/A) were obtained from Integrated Biotherapeutics (Rockville, MD). Control antibodies consisted of ChromPure goat IgG, whole molecule (catalog no. 005-000-003) and the conjugate PF–anti-goat IgG (derived from AffiniPure mouse anti-goat IgG (catalog no. 205-005-108), both from Jackson ImmunoResearch Laboratories, Inc.

##### Plasmonic Fluors (PFs)

PFs were produced by Auragent Biosciences, LLC, as previously described.^9^ Pan-filoviral antibodies and anti-goat IgG (AffiniPure mouse anti-goat IgG (H+L) (min X Hu, Ms, Rb Sr Prot), catalog no. 205-005-108, Jackson ImmunoResearch Labs) were conjugated to PF800, a product for the 800 nm channel. PF800/pan-filoviral antibodies were used as detection labels. PF800/anti-goat IgG was used as a control label.

##### PF-LFA Printing and Preparation

FF120HP Plus (25 mm width), a nitrocellulose-backed membrane bound to a 60 mm × 300 mm polystyrene card backing (catalog no. 10547129, Cytiva), was used for the preparation of the LFA strips. The test lines were applied by dispensing 0.5 mg/mL anti-EBOV capture antibody and 0.5 mg/mL anti-SUDV capture antibody with a contact reagent dispenser with a 5 mm spacing from each other. The control line was dispensed at a 5 mm distance from the test lines with 1 mg/mL of goat IgG (ChromPure Goat IgG, whole molecule, catalog no. 005-000-003, Jackson ImmunoResearch Labs). After being dispersed, the membranes were dried in a vacuum desiccator overnight.

For pre-incubation studies, a sample pad (Whatman Standard 14, Cytiva) blocked with 5% BSA, 5% Sucrose, 0.5% Tween 20, and 1X PBS and an absorbent pad (CF5, Cytiva) were assembled with the nitrocellulose membrane card, with an overlap of 2 mm, and then cut to strips with a width of 3 mm.

For strips with full-strip format, PF800/pan-filoviral antibodies were sprayed on a conjugate pad (Whatman Standard 14, Cytiva), and it was assembled with a sample pad (Fusion 5, Cytiva) blocked with 5% BSA, 5% sucrose, 0.5% Tween 20, and 1X PBS and an absorbent pad (CF5, Cytiva) along with a nitrocellulose membrane card, with an overlap of 2 mm, and then cut to strips with a width of 3 mm.

#### Reader Device

PF-LFAs were read on a custom-built, table-top reader constructed by Auragent Biosciences, LLC. Details can be found in previous literature.^9^ Briefly, the current table-top reader consists of an 80 mW, 785 nm diode laser (catalog no. Z80M18S3-F-785-pe, Zlaser) for excitation and an 832/37 nm emission filter (catalog no. 84-107, Edmund Optics).

#### Non-Human Primate (NHP) Serum Specimens

NHP specimens were obtained from a prior study of rhesus macaques challenged with EBOV and treated post-exposure with either a cocktail of three monoclonal antibodies (MB-03) or control.^28^ Specimens were identical to those used in a previous study utilizing a photonic resonator-based assay.^12^ USAMRIID standard procedures were used to process the specimens.^29^ RT-PCR status and days post-infection were known for each serum specimen received (Table S1-Sn).

All animal studies were performed under the approval of the local IACUC committees and were performed in compliance with the Animal Welfare Act and other federal statutes and regulations relating to animals. The USARMIID is accredited by the Association for Assessment and Accreditation of Laboratory Animal Care, International (AAALAC) and adheres to principles stated in the Guide for the Care and Use of Laboratory Animals, National Research Council. All challenge studies were conducted under maximum containment in an animal biosafety level (BSL)-4 facility at USAMRIID.

#### Pre-Incubation Studies

The specificity of SUDV and EBOV antibodies was assessed by incubation of samples within 10% pooled human serum diluted in 1X PBS with varying concentrations of spiked EBOV sGP and SUDV sGP. Lyophilized PF800/pan-filoviral antibodies and PF800/anti-goat IgG were incubated with the 10% human serum spiked with EBOV sGP and SUDV sGP for 0–60 min prior to delivery to the PF-LFA strip (60 μL of the pre-incubated sample was applied to the LFA strip). Each experimental parameter was completed for *n* = 1.

#### Non-human Primate Specimen Testing

NHP specimens were first diluted 10 times in 1X PBS and then incubated with lyophilized detection label and control label for 15 min prior to delivery to the PF-LFA strip (60 μL of the pre-incubated sample was applied to the LFA strip). Each sample was run *n* = 3, with the exception of Specimen 23 (*n* = 1) due to limited specimen availability.

## Supporting Information Available

Cross-reactivity between antibodies against EBOV sGP and SUDV sGP, response curves for the effect of serum percentage on PF-LFA performance, response curves for the direct application of specimens to the PF-LFA without pre-incubation, and data for NHP samples utilized in our study, including days post EBOV infection, PCR status, and level of sGP as determined by PF-LFAs (PDF)

## Abbreviations

BDBV: *Orthoebolavirus bundibugyoense*
CDC: Centers for Disease Control
DoD: United States Department of Defense
DRC: Democratic Republic of Congo
EBOV: *Orthoebolavirus zairense*
EVD: Ebolavirus disease
GP: glycoprotein
L: large protein
LFA: lateral flow assay
NAAT: nucleic acid amplification testing
NHP: non-human primate
NP: nucleoprotein
PF: plasmonic fluor
PF-LFA: plasmonic fluor lateral flow assay
RT-PCR: reverse transcriptase polymerase chain reaction
sGP: soluble glycoprotein
SUDV: *Orthoebolavirus sudanense*
TAFV: *Orthoebolavirus taiense*
VP40: viral protein 40
