# Supplementary material for: A Flexible, Quantitative Plasmonic-Fluor Lateral Flow Assay for the Rapid Detection of Orthoebolavirus zairense and Orthoebolavirus sudanense
Source: ACS Infect Dis. 2023 Dec 4;10(1):57–63. doi: 10.1021/acsinfecdis.3c00423 (PMC10788868; doi:10.1021/acsinfecdis.3c00423)
Supplement: Supplementary file 1 — id3c00423_si_001.pdf [file id3c00423_si_001.pdf]

Supporting Information

A flexible, quantitative plasmonic fluor lateral flow assay for the rapid detection of Orthoebolavirus zairense and Orthoebolavirus sudanense

Authors: Abraham J. Qavi[1,*], Qisheng Jiang[2], M. Javad Aman[3], Hong Vu[3], Larry Zetlin[4], John M. Dye[5], Jeffrey W. Froude[6], Daisy W. Leung[7], Frederick Holtsberg[3], Scott L. Crick[2], Gaya Amarasinghe[8,*]

Affiliations:
1. Department of Pathology and Laboratory Medicine, University of California, Irvine CA 92617, USA
2. Auragent Bioscience, St. Louis, Missouri 63108, United States
3. Integrated Biotherapeutics, Rockville, MD 20850, USA
4. Mapp Biopharmaceutical, Inc., San Diego, CA 92121, USA
5. United States Army Medical Research Institute of Infectious Diseases, Fort Detrick, MD 21702, USA
6. United States Army Nuclear and Countering Weapons of Mass Destruction Agency, Fort Belvoir, VA, 22060, USA
7. Department of Medicine, Washington University School of Medicine, St. Louis, MO 63110, USA
8. Department of Pathology & Immunology, Washington University School of Medicine, St. Louis, MO 63110, USA

*** Corresponding Author Information:** Abraham Qavi, aqavi@uci.edu; Gaya K. Amarasinghe, gamarasinghe@wustl.edu.

Contents: 7 pages, 3 figures, 1 table

Overview

Figure S1-Sn. Limited cross-reactivity between antibodies against EBOV sGP and SUDV sGP.

Figure S2-Sn. Increasing serum percentages lead to decreased performance of PF-LFAs.

Figure S3-Sn. Direct application of specimens to the PF-LFA without pre-incubation.

Table S1-Sn. Non-human primate serum specimens utilized in our study.

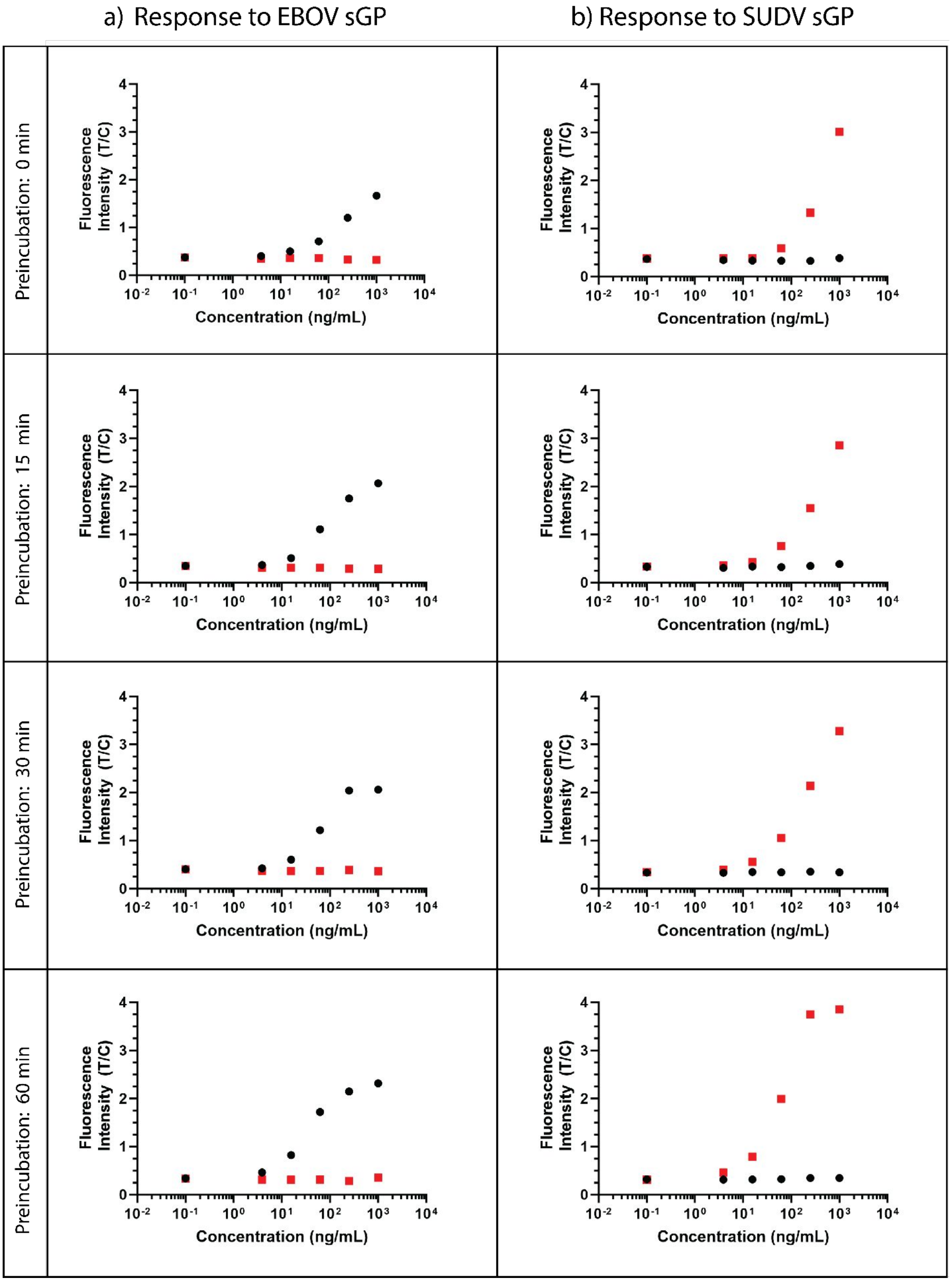

a) Response to EBOV sGP
b) Response to SUDV sGP
Preincubation: 0 min
Preincubation: 15 min
Preincubation: 30 min
Preincubation: 60 min
Fluorescence Intensity (T/C)
Concentration (ng/mL)

Figure S1-Sn. Limited cross-reactivity between antibodies against EBOV sGP and SUDV sGP. PF-LFA strips exposed to varying concentrations of (a) EBOV sGP and (b) SUDV sGP in 10% pooled human serum. Each row represents a different amount of pre-incubation time, from 0 to 60 min. (●) = response on EBOV stripes, (■) = response on SUDV stripes. The signal intensity, T/C, indicates the fluorescent intensity of the test line (T) divided by the control line (C). The x-axis is concentration of sGP (ng/mL).

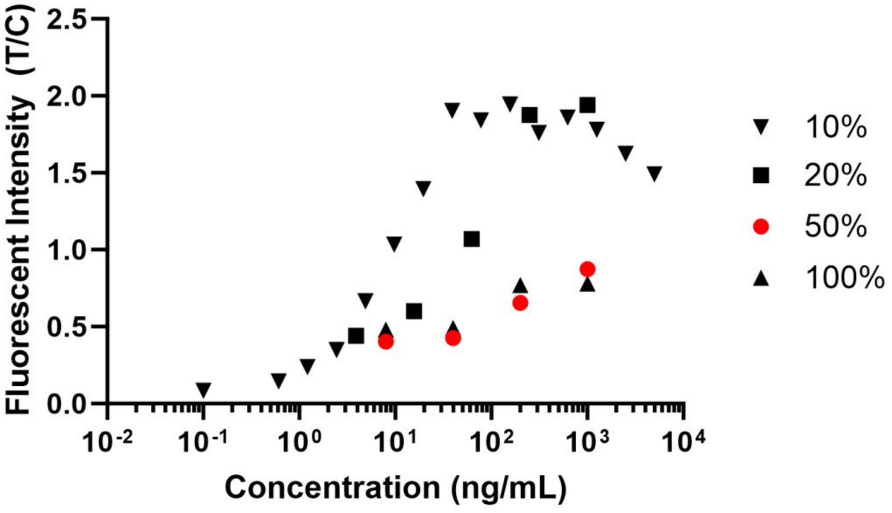

Figure S2-Sn. Increasing serum percentages lead to decreased performance of PF-LFAs. Increased serum percentages applied to the PF-LFA lead to a decrease in signal and performance. Each data point was performed with n = 1, with the exception of 10% serum, performed with n = 2.

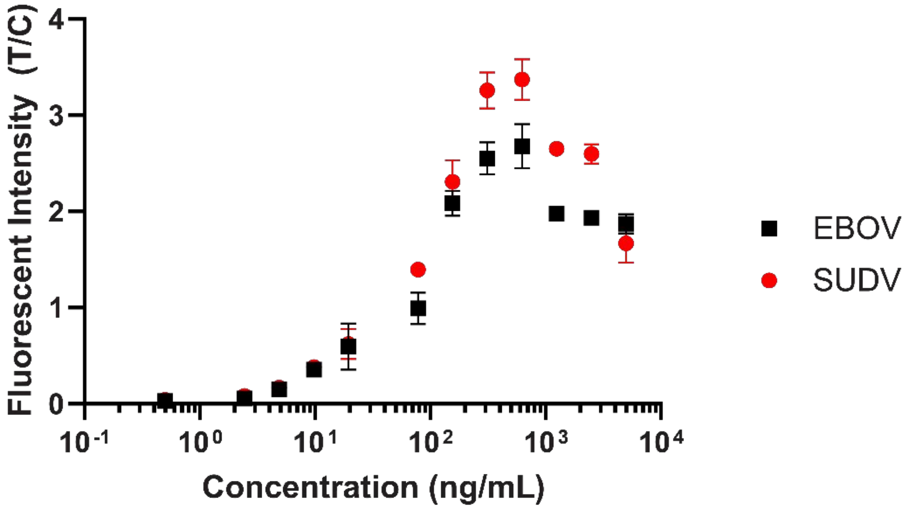

Figure S3-Sn. Direct application of specimens to the PF-LFA without pre-incubation. Calibration curves with no pre-incubation and direct application of spiked samples to the strip. Limit of detection for EBOV and SUDV sGP were 2.15 ng/mL and 1.07 ng/mL, respectively. Error bars represent the standard deviations for n = 2.

Specimen	Days Post Infection	PCR Status	Concentration of sGP (ng/mL)
1	5	Positive	10.71
2	7	Positive	80.72
3	9	Negative	42.33
4	9	Negative	52.96
5	7	Negative	106.98
6	7	Negative	78.87
7	9	Positive	≥400
8	7	Positive	239.2
9	11	Negative	119
10	6	Positive	≥400
11	8	Positive	≥400
12	8	Positive	≥400
13	9	Positive	≥400
14	8	Negative	≥400
15	9	Negative	≥400
16	10	Positive	≥400
17	9	Positive	≥400
18	8	Positive	≥400
19	11	Positive	≥400
20	10	Negative	≥400
21	9	Positive	≥400
22	10	Positive	≥400
23	11	Positive	≥400
24	11	Positive	≥400
25	10	Positive	≥400
26	11	Positive	≥400
27	6	Positive	≥400
28	7	Positive	≥400
29	8	Positive	≥400
30	9	Positive	≥400

Table S1-Sn. Non-human primate serum specimens utilized in our study. Included are days post EBOV infection, PCR status as determined by USAMRIID testing, and the concentration of sGP determined by the PF-LFA. Results with ≥400 ng/mL represent signals that saturated the response.
